# Prophylactic infusion of norepinephrine does not affect the rostral spread of spinal anesthesia in pregnancy: a prospective, randomized, double-blinded study

**DOI:** 10.3389/fphar.2023.1340452

**Published:** 2024-01-09

**Authors:** Yu-Fang Dong, Jing Qian, Jing Wang, Li-Zhong Wang, Xing-Hua Qian, Fei Xiao

**Affiliations:** Department of Anesthesia, Jiaxing University Affiliated Women and Children Hospital, Jiaxing, China

**Keywords:** norepinephrine, spinal anesthesia, hypotension, pregnancy, bupivacaine

## Abstract

**Background:** The infusion of phenylephrine to prevent spinal-induced hypotension (SIH) in cesarean delivery may decrease the rostral spread of a spinal local anesthetic. We hypothesized that infusion of norepinephrine may decrease the rostral spread of spinal anesthesia, similar to that caused by phenylephrine. The aim of this study was to compare the block height of spinal anesthesia in the presence or absence of norepinephrine infusion administered to prevent SIH during cesarean delivery.

**Methods:** Eighty patients were enrolled and allocated into groups receiving a norepinephrine infusion (group N) or saline infusion (group C). After intrathecal injection of hyperbaric bupivacaine 10 mg, the block height for cold and pinprick sensation was checked 10 and 20 min after the injection. The demographic characteristics, spinal anesthesia, side effects, and neonatal outcomes were also recorded.

**Results:** The block height for cold and pinprick sensation was similar between the two groups, although the incidence of hypotension was significantly lower (*p <* 0.00) in group N than in group C. Systolic blood pressure was also more stable in group N than in group C, with the incidence of interventions being significantly lower in group N. There was no significant difference in patient satisfaction between the two groups.

**Conclusion:** Evidence from this study suggested that prophylactic norepinephrine infusion does not reduce the rostral spread of spinal anesthesia in pregnancy. We suggest that it is not necessary to increase the dose of an intrathecal local anesthetic for cesarean delivery when prophylactic norepinephrine is administered.

**Clinical Trial Registration**: https://www.chictr.org.cn/bin/project/edit?pid=152899, identifier [ChiCTR2200057439].

## Introduction

Hypotension is a common side effect of spinal anesthesia in pregnant women undergoing cesarean delivery Chooi et al. (2020); Massoth et al. (2020). To prevent spinal-induced hypotension (SIH) and its associated intraoperative nausea and vomiting (INOV), prophylactic infusion of either phenylephrine or norepinephrine is an ideal strategy and well-accepted technique, used often during cesarean delivery Chooi et al. (2020); Fu et al. (2020); Massoth et al. (2020); Ngan Kee et al. (2020); Wei et al. (2020); Xiao et al. (2020). However, infusion of phenylephrine to prevent SIH is associated with a decrease in the rostral spread of spinal anesthesia in pregnant women undergoing cesarean delivery Cooper and Mowbray (2003); Cooper et al. (2004). This decrease is possibly due to the alpha agonist effects of phenylephrine on epidural venous tone, although the exact mechanism is still unknown Cooper and Mowbray (2003); Cooper et al. (2004). Currently, there is also no evidence on the effects of norepinephrine on epidural venous tone. Therefore, we hypothesized, preventive norepinephrine infusion may also decrease the rostral spread of spinal anesthesia, similar to that observed with phenylephrine. Accordingly, we designed a study to determine the effect of preventive norepinephrine infusion on the spread of spinal anesthesia. The null hypothesis of this study was that norepinephrine infusion does not affect the spread of spinal anesthesia.

## Methods

### Ethics

The study was approved by the Jiaxing University Affiliated Women and Children Hospital’s Institutional Review Board (IRB KY-2022-05, date of approval: 19 January 2022). All parturients recruited in this study signed written, informed consent. We registered the clinical trial in the Chinese Clinical Trials Registry at https://www.chictr.org.cn/(ChiCTR2200057439, principal investigator: Yu-Fang Dong, date of registration: 12 March 2022) before enrollment of the parturients, which was initiated on 15 March 2022, and concluded on 21 June 2022.

### Design

The study was a prospective, randomized, double-blind design.

### Patients and setting

Consecutive parturients who met the following inclusion criteria and were scheduled for elective cesarean delivery were enrolled in the study. The inclusion criteria were as Society of Anesthesiologists (ASA) physical status < Ⅲ, singleton pregnancy at term (37 weeks ≤ gestation age ≤41 weeks), age 20–40 years, height 158–170 cm, and body mass index (BMI) ≤ 35 kg/m^2^. The exclusion criteria were preeclampsia or preexisting hypertension, preexisting or gestational diabetes, intrauterine growth retardation or preterm delivery, allergic to norepinephrine and local anesthetics, and any contraindications to spinal or epidural anesthesia, including a bleeding disorder, local infection, or intracranial hypertension.

Randomization of the parturients was carried out by a research assistant who used a numbered sheet generated by an online randomization generator (https://www.random.org/sequences/) to allocate them to receive an infusion of either norepinephrine (group N) or saline (group C). The randomized scheme was then concealed in sequentially numbered, opaque envelopes, with one opened for each patient enrolled.

### Study protocol

All parturients enrolled in the study fasted without solid food for 8 h and water for 2 h. No premedication was administered. Upon arrival in the operating room, standard monitoring for vital signs was applied for continuous measurement, including non-invasive blood pressure, electrocardiogram, and pulse oximetry. The baseline values for systolic blood pressure (SBP) and heart rate (HR) were calculated as the mean of three continuous measurements of SBP and HR at 3-min intervals, 5 min after the patient had become calm. Peripheral intravenous access in the left upper limb was then created through an 18-G trocar. No prehydration was given.

Combined spinal-epidural anesthesia was achieved via a needle-through-needle technique at the ascertained L3-4 vertebral interspace, which was located by ultrasound assessment, with the patient in the left lateral position under regional anesthesia. The epidural space was assessed by the loss-of-resistance of saline (<2 mL) using an 18-gauge Tuohy. A 25-gauge Whitacre needle was then passed through the Tuohy needle to reach the subarachnoid space. After the emergence of clear cerebrospinal fluid (CSF), 10 mg of hyperbaric bupivacaine (2 mL) was injected into the subarachnoid space over 15 s, with the bevel rostral. Before removal of the Whitacre needle, gentle aspiration was applied by the syringe to ensure that CSF could be withdrawn, which indicated successful delivery of the intrathecal medications had occurred. An epidural nylon catheter with multiple holes (three holes) was then inserted 3–4 cm into the epidural space.

Immediately after the intrathecal injection, an infusion of 0.1 ㎍/kg/min norepinephrine, or saline, was prepared by a fixed anesthesia assistant who knew the patients’ grouping but did not participate in patient care and data collection. The infusions were administered using a syringe pump. Meanwhile, a coload of 10 mL/kg/h of warmed Lactate Ringer solution was infused. Hypotension was defined as an absolute SBP value <90 mmHg or a decrease ≥20% in the baseline value, hypertension as a SBP ≥120% of baseline value, and bradycardia as an HR < 50 bpm. According to the study protocol, we treated hypotension accompanied by an increase in HR with 50 ㎍ of phenylephrine, hypotension occurring with bradycardia with 0.5 mg atropine and/or 6 mg ephedrine, and hypertension by stopping the infusion of norepinephrine and restarting when SBP was <120% of the baseline value.

### Measurements

The primary outcome in this study was block height measured 10 and 20 min after the intrathecal injection. Alcohol wipes were used to assess cold sensation, with the patients asked to report changes in temperature perception. A blunted epidural needle was used to assess pinprick sensation and the patients were required to report if the blunt needle caused pain, felt sharp, or both. The secondary outcomes of this study were: side effects such as hypotension; hypertension; bradycardia; shivering; nausea and vomiting; physician interventions including treatment of hypotension, hypertension, or bradycardia; surgical data including duration of surgery and duration from intrathecal injection to infant delivery; patient satisfaction was evaluated immediately following the completion of surgery using a 0–5 scale, where 0 signifies dissatisfaction and five represents the highest level of satisfaction; and neonatal outcomes including 1, 5 min Apgar score and umbilical arterial pH value; and patient satisfaction (0–5, where 0 presents most unsatisfied, 5 presents most satisfied). Demographic characteristics including maternal age, height, weight, and gestational age were also recorded.

### Calculation of sample size

Group sample sizes of 24 and 24 achieve 90% power to show a difference in means when there is a difference of 2.0 (significance for clinical practice) between the null hypothesis mean difference of 0.0 and the actual mean difference of 2.0 at the 0.050 significance level (alpha) using a two-sided Mann-Whitney-Wilcoxon Test. These results are based on 2000 Monte Carlo samples from the null distributions: Normal (M0 S) and Normal (M0 S), and the alternative distributions: Normal (M0 S) and Normal (M1 S). To account for possible dropouts, we enrolled 40 parturients for each group in this study.

### Statistical analysis

The Kolmogorov-Smirnov test was used to assess the distribution of the univariable data. Normally distributed data, including the demographic characteristics, surgery time, and umbilical arterial pH value were expressed as mean ± standard deviation (SD), with differences analyzed using Student’s t*-*test. Non-normally distributed data, such as block height, were expressed as median (range) and were analyzed using the Mann-Whitney U test. The incidence of side effects was expressed as a number (incidence) and tested using Fisher’s exact test. A *p*-value <0.05 was regarded as statistically significant (two-sided). The analyses were performed using GraphPad Prism version 5.0 (GraphPad Software Inc., San Diego, CA, United States).

## Results

**FIGURE 1:**
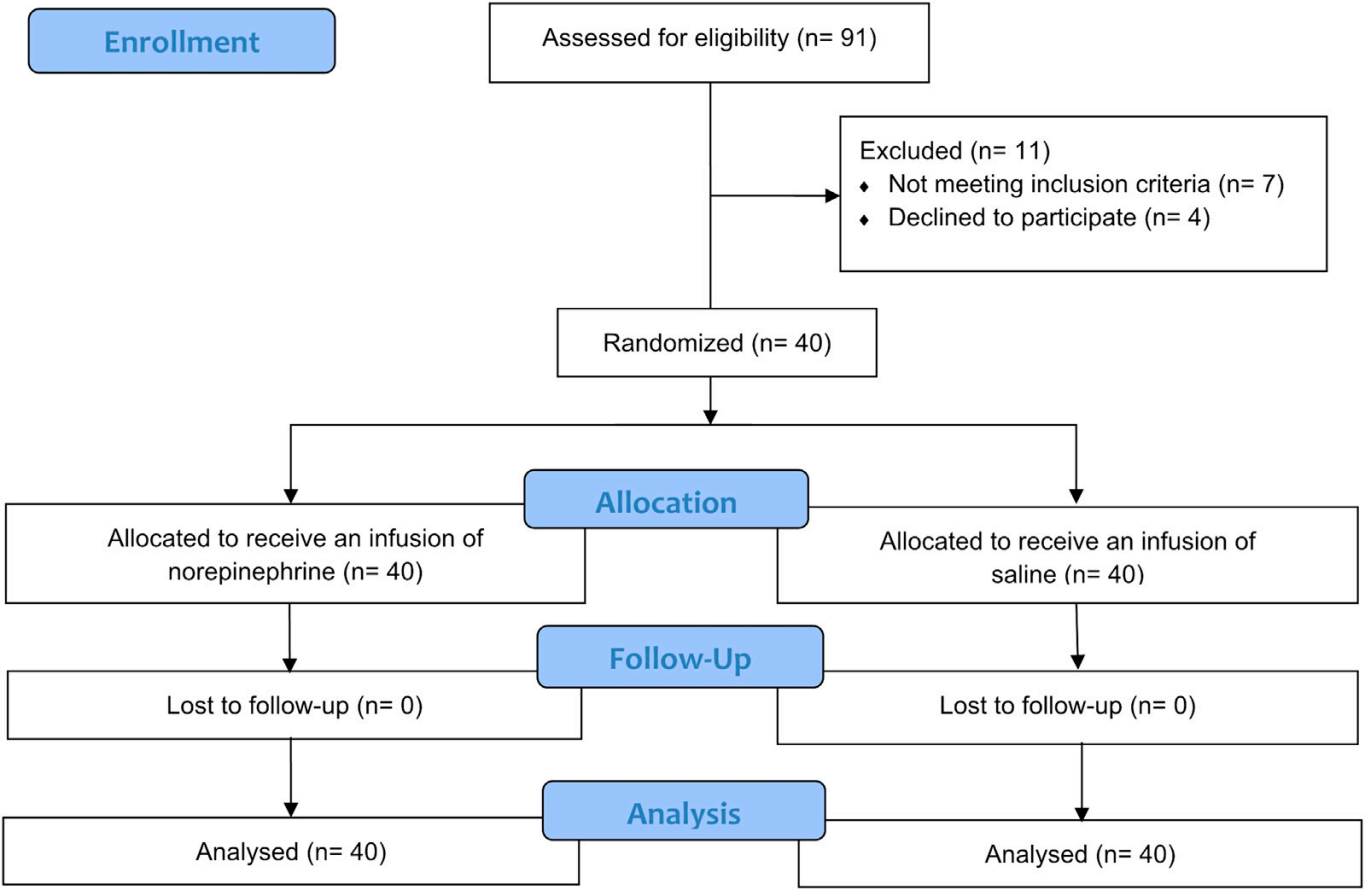
CONSORT flow diagram.

A total of 91 patients were enrolled for the assessment of eligibility. Of these 91 patients, 7 did not meet the inclusion criteria and 4 declined to participate, leaving 80 patients in the final analysis. The patients were then randomized into one of the two study groups. The CONSORT flow diagram is shown in Figure 1. As shown in Table 1, there was no significant difference in the demographic characteristics and surgical data between the two groups.

**TABLE 1:** Parturient Characteristics Data are presented as Mean ± standard deviation (SD).

Groups	Norepinephrine group (*n* = 40)	Saline group (*n* = 40)	*p*-Value
Age, yr	32.3 ± 4.7	31.5 ± 4.6	0.457
Height, cm	161.1 ± 3.9	160.2 ± 2.6	0.237
Weight, kg	71.5 ± 9.9	71.3 ± 7.6	0.537
Gestational age, wk	39.8 ± 0.8	39.0 ± 0.8	0.704
Surgery time, min	51 ± 10	48 ± 11	0.338
Fetal delivery time, min	15.9 ± 4.3	16.2 ± 4.4	0.779

**FIGURE 2:**
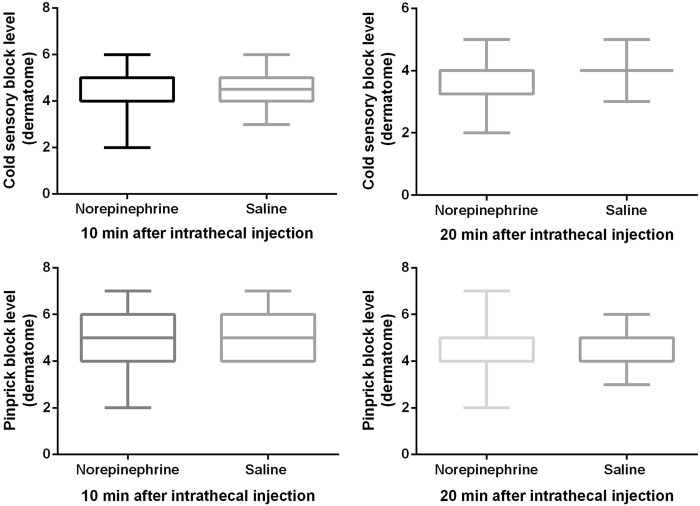
Block height for cold and pinprick sensation. The cold sensory block level was 4 (4, 5) vs. 4.5 (4, 5) at 10 min, and 4 (3, 4) vs. 4 (4, 4) at 20 min after intrathecal injection. The pinprick sensory block level was 5 (4, 6) vs. 5 (4, 6) at 10 min, and 4 (4, 5) vs. 4 (4, 5) at 20 min after intrathecal injection. Boxplots show median, 10th, 25th, 75th, and 90th percentiles. There was no significant difference between the two groups (Mann-Whitney *U* test, all *p* values > 0.05).

Block heights were similar between the two groups for cold and pinprick sensation 10 and 20 min after the intrathecal injection (Figure 2). With the exception of seven patients in group N and nine patients in the group C whose block height for cold sensation reached T5 20 min after the injection, all the remaining patients recorded a response ≥ T4. All 12 patients did not require an epidural top-up. One patient in group N whose block height for pinprick sensation only reached T7 received an epidural injection of 5 mL of 2% lidocaine 20 min after the intrathecal injection. No patient in group C required this extra treatment. Only one patient in group C complained of being uncomfortable and felt pain during surgery, and was therefore administered 10 mL of 2% lidocaine epidurally. There was no significant difference (*p* = 0.26) in patient satisfaction between group N (5, (4-5)) and group C (5, (2-5)), with no patient requiring general anesthesia.

Subgroup (patients who used phenylephrine to treat hypotension were excluded) analysis showed that there was no difference in the cold or pinprick sensation for 10, and 20 min after intrathecal injection between the two groups, which are shown in Table 2.

**TABLE 2:** Comparison of the sensory block for subgroup.

	Group norepinephrine (*n* = 36)	Group saline (*n* = 17)	*p*-Value
Lost cold sensation at 10 min after spinal injection	4 (3, 6)	5 (2, 6)	0.490
Lost pinprick sensation at 10 min after spinal injection	5 (2, 7)	5 (4, 7)	0.358
Lost cold sensation at 20 min after spinal injection	4 (2, 5)	4 (3, 5)	0.211
Lost pinprick sensation at 20 min after spinal injection	4 (2, 7)	5 (3, 6)	0.117

Data are presented as Median (range).

**FIGURE 3:**
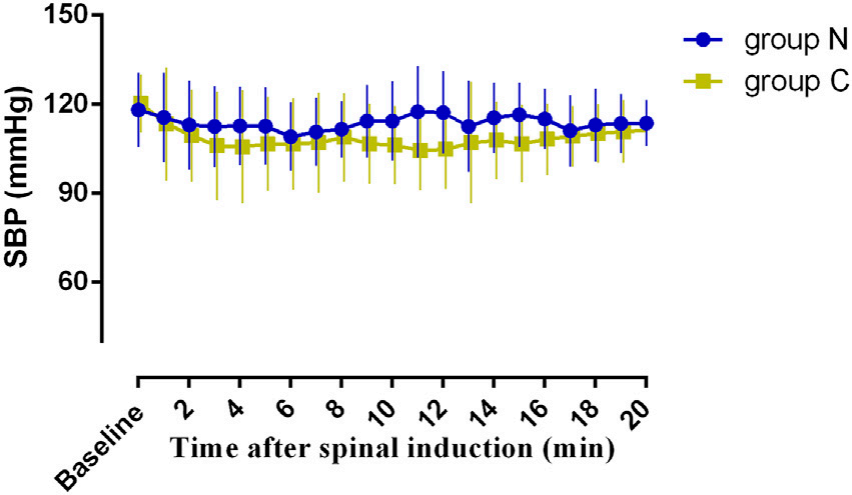
Systolic blood pressure 20 min after the intrathecal injection. The area under the curve (mean ± SD) was not significantly different between the two groups (2273 ± 40, and 2161 ± 46 min·mmHg in group N and group C, *p* < 0.05).

The incidence of side effects is shown in Table 3. The incidence of hypotension was significantly lower (*p <* 0.001) in group N (10%) than in group C (57.5%). The incidence of nausea and vomiting was lower (*p* = 0.048) in group N than in group C. As shown in Figure 3, SBP measured at 1-min intervals in the first 20 min after the intrathecal injection, was closer to the baseline value in group N than in group C, with no significant difference in the area under the curve between the two groups (*p >* 0.05). Two patients in group N experienced reactive hypertension, although there was no significant difference in incidence between the two groups (*p =* 0.494). There was also no significant difference in the incidence of shivering between the two groups. The incidence of physician interventions was significantly lower in group N than in group C, while patient satisfaction showed no significant difference between the two groups.

**TABLE 3:** Side effects, physician interventions, patient satisfaction, and neonatal outcomes.

	Group norepinephrine (*n* = 40)	Group saline (*n* = 40)	*p*-Value
Hypotension	4 (10.0%)	23 (57.5%)	<0.001
Hypertension	2 (5.0%)	0 (0%)	0.494
Nausea	2 (5%)	6 (15%)	0.136
Vomiting	1 (2.5)	3 (7.5)	0.288
Bradycardia	2 (5%)	6 (15%)	0.136
Shivering	3 (7.5%)	6 (15%)	0.288
Incidence of patients need physician interventions	6 (15%)	24 (60.0%)	<0.001
Frequency in patients need physician interventions	1 (1, 1)	2 (1, 3)	0.003
Patient satisfaction	5 (4-5)	5 (2-5)	0.26
1-min Apgar score	9.65 ± 0.58	9.57 ± 0.68	0.60
5-min Apgar score	9.70 ± 0.52	9.70 ± 0.46	1.0
umbilical arterial pH value	7.27 ± 0.08	7.28 ± 0.07	0.56

Data were presented as number (incidence) or median (range), as appropriate.

The neonatal outcomes are summarized in Table 3 that shows there were no significant differences in the 1 and 5-min Apgar scores and umbilical arterial pH value between the two groups.

## Discussion

The results of this study suggested that norepinephrine infusion to prevent spinal-induced hypotension in cesarean delivery does not affect the rostral spread of spinal anesthesia. This result underlies that it is not necessary to increase the dose of intrathecal bupivacaine in order to reach a satisfactory level of anesthesia when prophylactic norepinephrine infusion is used to manage post-spinal blood pressure. Previous studies have suggested that when phenylephrine is used as a preventive infusion in cesarean delivery this may lead to a decrease in the rostral spread of spinal anesthesia. Cooper et al. (2004) Recently, it has been reported that norepinephrine has sufficient potency to act as an alternate to phenylephrine in obstetric anesthesia. Ngan Kee et al. (2015); Mercier et al. (2019); Theodoraki et al. (2020) However, there is only limited data on the effect of norepinephrine on intrathecal anesthetic spread. The strength of the current study was that it provided evidence on the effect of norepinephrine in this context.

The requirement for an intrathecal local anesthetic for pregnant women undergoing cesarean delivery is lower than that for non-pregnant women undergoing surgery under spinal anesthesia. This is due to dilation of the epidural veins that leads to a reduction in lumbosacral CSF volume. Cooper et al. (2004) Similarly, evidence has shown that an epidural injection of 10 mL of saline slightly increases the volume of the epidural space and subsequently decreases the volume of lumbosacral CSF, thereby increasing the rostral spread of spinal anesthesia. Stienstra et al. (1999)Therefore, we assumed that both norepinephrine and phenylephrine may reduce the spread of spinal anesthesia by contracting the epidural veins and offsetting the physiological expansion associated with pregnancy.

However, data from the current study showed no decremental spread in spinal anesthesia, a result which is contrary to our hypothesis and different from those of a previous study Cooper et al. (2004). After carefully reviewing the protocols of Cooper (Cooper et al., 2004) and our studies, we found the specific gravity of the local anesthetics used in the two studies were different (10 mg of plain levobupivacaine in Cooper’s study compared to 10 mg of hyperbaric bupivacaine in the current study). We concluded that this was the most likely reason for the different findings between the two studies. In the supine position, a hyperbaric local anesthetic solution spreads rostrally under the action of gravity, until the thoracic curve of the vertebral canal is reached and limits further spread Hocking and Wildsmith (2004); Loubert et al. (2011). In contrast, the spread of a plain anesthetic solution is dependent mainly on the flow of CSF rather than restricted by a physiological structure. Clinical trials also provided evidence that intrathecal plain bupivacaine was more effective for rostral spread than that of hyperbaric bupivacaine following epidural volume extension Tyagi et al. (2008). We therefore consider that the difference in CSF flow caused by vasopressors has less effect on the rostral spread of hyperbaric bupivacaine than that caused by plain levobupivacaine, due to the spread of hyperbaric bupivacaine being more dependent on the effect of gravity.

Another mechanism causing this phenomenon may be the different pressures in the epidural space caused by phenylephrine and norepinephrine. In their study, Cooper (Cooper et al., 2004) used 67 ㎍/min of phenylephrine, whereas we used 0.1 ㎍/kg/min of norepinephrine, equivalent to 7.1 ㎍/min based on the mean body weight of group N (71 kg). Evidence shows that the efficacy ratio for continuous infusion of norepinephrine to phenylephrine is approximately 1:6 Qian et al. (2022). Therefore, 67 ㎍/min of phenylephrine has a greater vasoconstrictive effect than 7.1 ㎍/min of norepinephrine (equivalent to 42.6 ㎍/min of phenylephrine). This results in lower epidural pressure which may reduce the rostral spread of the intrathecal local anesthetic. Further clinical investigations are therefore warranted to confirm the equivalent vasopressor effect of spinal spread.

It could be argued that our use of phenylephrine to treat hypotension could possibly confound our results, although there was no evidence to show that an intravenous bolus of rescued phenylephrine affected the rostral spread of spinal anesthesia. Taking this into account, we conducted a subgroup analysis, in which patients who suffered hypotension and received phenylephrine were excluded. The analysis showed no difference in cold or pinprick sensation at 10, and 20 min after the spinal injection between the two groups. Despite this, further studies with large simple size are warranted.

That we chose 0.1 ㎍/kg/min of norepinephrine as the infusions rate was based on the results of our previous studies, in which we found 0.1 ㎍/kg/min of norepinephrine would be an ideal initial and safe dose for preventing SIH during cesarean delivery for maintaining the haemodynamics and without any peripheral venous complications Xu et al. (2021). Although of that, there remain concerns regarding the safety of peripheral administration of a norepinephrine infusion using such a large dose. However, prior data have shown that peripheral venous delivery of norepinephrine is safe and feasible. A recent large multicenter study of non-obstetric patients showed no significant correlation between the use of peripheral norepinephrine infusions and adverse events Pancaro et al. (2020). Similarly, the absence of peripheral venous complications in our study suggests that the administration of norepinephrine via the peripheral venous route is both safe and feasible. Nevertheless, we recommend peripheral administration of norepinephrine via a large vein with concurrent intravenous fluid infusion through the same vein.

Similar to previous studies (Ngan Kee et al., 2004; Siddik-Sayyid et al., 2014; George et al., 2018),our results showed that prophylactic vasopressor infusion to prevent SIH was superior to an intravenous bolus administration of vasopressor to treat hypotension, because of more stable hemodynamics state (Figure 3) and a lower incidence of hypotension. Our study also showed a low incidence of reactive hypertension with an infusion of 0.1 ㎍/kg/min norepinephrine, a result which was in accordance with that of our previous study Xu et al. (2021). The higher incidence and frequency of physician intervention required in the saline group suggested that without a preventive strategy to manage post-spinal hemodynamics, this would inevitably result in a heavier workload for obstetric anesthesia care providers. Although patients without prophylactic norepinephrine experienced a high incidence of hypotension, but there was no significant difference in patient satisfaction between the two groups due to the comparable incidence of nausea and vomiting, resulting from prompt observation and management.

We acknowledge there are several limitations in the current study. First, phenylephrine was used to treat hypotension in the control group, which would have influenced the results of the rostral spread observed with intrathecal hyperbaric bupivacaine. However, there was no evidence to show that a bolus of phenylephrine could influence spinal spread. We choose phenylephrine rather than ephedrine to treat hypotension because it is superior for maintaining the pH value of the umbilical artery blood. Further studies are therefore needed to compare the direct effects of infusion of phenylephrine or norepinephrine on the rostral spread of spinal medications. Second, the sample size calculated for the current study was for primary outcomes, but not for secondary outcomes. There are inevitable statistical errors that may occur and therefore the conclusions derived from these results can only be regarded as explorative for clinical practice. Third, no objective indicators were used to assess block height in the current study. Therefore, the assessment of block height was subjective, with different people having different standards for their post-spinal perception of cold and pinprick sensation. This will have inevitably affected the results. Finally, the observed side effects in this study may not accurately reflect the impact on neonatal outcomes. Nonetheless, studies conducted in this context have demonstrated that similar to phenylephrine, norepinephrine is both safe and effective for neonates, even in women with fetal compromise Mohta et al. (2022).

In summary, this study suggested that prophylactic infusion of norepinephrine does not reduce the rostral spread of intrathecal hyperbaric bupivacaine in pregnancy, and that it is not necessary to increase the dose of the intrathecal local anesthetic. However, further studies are warranted to compare the direct effects of phenylephrine and norepinephrine infusions on the rostral spread of spinal medications and intrathecal dose requirement of local anesthetic.

## Data Availability

The original contributions presented in the study are included in the article/supplementary material, further inquiries can be directed to the corresponding authors.
